# Compiling probabilistic, bio-inspired circuits on a field programmable analog array

**DOI:** 10.3389/fnins.2014.00086

**Published:** 2014-05-07

**Authors:** Bo Marr, Jennifer Hasler

**Affiliations:** ^1^Raytheon, Space and Airborne SystemsManhattan Beach, CA, USA; ^2^Georgia Institute of TechnologyAtlanta, GA, USA

**Keywords:** FPAA, probabilistic hardware, reconfigurable analog, dymamical system, bio-inspired, Hardware accelerator, biological computational model, probability theory

## Abstract

A field programmable analog array (FPAA) is presented as an energy and computational efficiency engine: a mixed mode processor for which functions can be compiled at significantly less energy costs using probabilistic computing circuits. More specifically, it will be shown that the core computation of any dynamical system can be computed on the FPAA at significantly less energy per operation than a digital implementation. A stochastic system that is dynamically controllable via voltage controlled amplifier and comparator thresholds is implemented, which computes Bernoulli random variables. From Bernoulli variables it is shown exponentially distributed random variables, and random variables of an arbitrary distribution can be computed. The Gillespie algorithm is simulated to show the utility of this system by calculating the trajectory of a biological system computed stochastically with this probabilistic hardware where over a 127X performance improvement over current software approaches is shown. The relevance of this approach is extended to any dynamical system. The initial circuits and ideas for this work were generated at the 2008 Telluride Neuromorphic Workshop.

## 1. Introduction

Due to the large computational efficiency gap that is theorized between classic digital computing and neuromorphic style computing, particularly in biological systems, this work seeks to explore the potential efficiency gains of a neuromorphic approach using stochastic circuits to solve dynamical systems.

There is wide demand for a technology to compute dynamical systems much more efficiently with some recent examples being to calculate quantum equations to aid in the development of quantum computers or in the search for new meta-materials and pharmaceuticals using high computational throughput search methods for new meta-compounds. Standard digital computers—even super computers—have proven to be inefficient at these tasks limiting our ability to innovate here.

Stochastic functions can be computed in a more efficient way if these stochastic operations are done natively in probabilistic hardware. Neural connections in the cortex of the brain is just such an example and occur on a stochastic basis (Douglas, 2008). The neurotransmitter release is probabilistic in regards to synapse firings (Goldberg et al., 2001) in neural communication. Most germaine to this work, many chemical and biological reactions occur on a stochastic basis and are modeled here via probabilistic circuits compiled on a reconfigurable field programmable analog array (Gillespie, 1976).

Gillespie effectively showed that molecular reactions occur probabilistically and gave a method for translating a system of *N* chemical equations, normally specified by deterministic differential equations, into a system of probabilistic, Markov processes. This will be the dynamic system described and computed herein. It has been shown that in a system with a sufficiently small number of molecules, the stochastic method is *more accurate* than its deterministic counterpart. It was further proven that in the thermodynamic limit (large number of molecules) of such a system, the deterministic and stochastic forms are mathematically equivalent (Oppenheim et al., 1969; Kurtz, 1972).

Mathematical results will be extended from Gillespie's algorithm to show that *any dynamical system* can be computed using a system of stochastic equations. The efficiency in computing such a system is greatly increased by a direct, probabilistic hardware implementation that can be done in the analog co-processor we present. However, *how* to express a general dynamical system stochastically will not be discussed, only that a formulation exists that is more efficient when computed with natively probabilistic hardware.

Several encryption algorithms and other on-chip solutions for a uniformly random number generator in hardware have been shown for microprocessors (Ohba et al., 2006). Generating static Bernoulli trials, where a 1 is generated with fixed probability *p* and 0 is generated with fixed probability 1 − *p*, were proposed using amplified thermal noise across digital gates and is also not a novel concept, but the work in which this concept was described was not fabricated, measured, or applied in hardware, and only existed in theory (Chakrapani et al., 2006). Static probabilities, or those with a fixed *p*-value, will not allow the performance gains that are possible in many stochastic systems because without dynamic *p*-values stochastic processes cannot be fully realizable in hardware.

There has been a hardware solution proposed for dynamic Bernoulli trial generators, where the probability *p* can be dynamically reconfigured via reprogramming a floating gate transistor (Xu et al., 1972). While this latter work illustrates a note-worthy solution and is a unique implementation to provide dynamic probability adjustment, there is an overhead cost in terms of time to readjust the probability *p* due to the nature of floating gate programming, which were not designed in that work for the continuous updates that are required for the applications presented here.

The topic of generating stochastic variables with hardware circuits has also been addressed previously in Genov and Cauwenberghs (2001), but not in this manner. We make a contribution to the literature by showing that not only can we produce stochastic variables, but we can tune the probability of these stochastic variables in real time through a software controllable input to the Bernoulli trial generator circuit via the FPAA. Further, we show how an array of these can be compiled on hardware and where outputs are input to a priority encoder to create an exponentially random variable for the first time known to the authors. Finally, this paper shows how the FPAA, with tunable stochastic variables which can be dynamically tuned in real time, results in significant performance gains of 127X for computing dynamical systems.

In short, this paper will present several novel contributions including

- A novel circuit for fast dynamic Bernoulli random number generation.
- A compiled chaos circuit to generate environment independent probabilistic variables.
- A novel circuit for fast dynamic exponentially distributed random number generation.
- Analysis of the performance gains provided by the latter circuits over current methods for Gillespie's algorithm that apply to many biological applications and stochastic applications in general.
- Extension of the latter methods for applicability to any dynamical system and the result that all dynamic systems calculated stochastically require exponentially distributed random numbers.
- A method for going from concept to circuit measurements in 2 weeks using a novel reconfigurable chipset developed in part by the authors.

Section 2 introduces the technology behind building probabilistic function generators in hardware using thermal noise characteristics. Section 3 reviews implementation of dynamical systems in general and specifically Gillespie's Algorithm to give a context for why these circuits are important. Section 4 will review the chipset that was built in part by the authors and how it can used for faster stochastic computation. Section 5 will discuss the hardware results and experimental measurements. Section 6 will conclude the paper and discuss future directions.

## 2. Extremely efficient stochastic circuits

Stochastic computation has shown to be a powerful tool to compute solutions to systems that would otherwise require complex continuous-time differential equations. However, the efficacy of stochastic methods are lost if complex computations are needed to produce the digital representation of these stochastic results.

We present several circuits to solve these issues that can uniquely be compiled in analog technology, and thus our FPAA co-processor.

### 2.1. A programmable thermal noise circuit

Thermal noise is a well-defined, well-behaved phenomenon that we show can be used as a computational resource within the FPAA fabric. This work will show that it can be used not only as a resource for random number generation, but for the generation of arbitrarily complex probabilistic functions.

**Figure 1:**
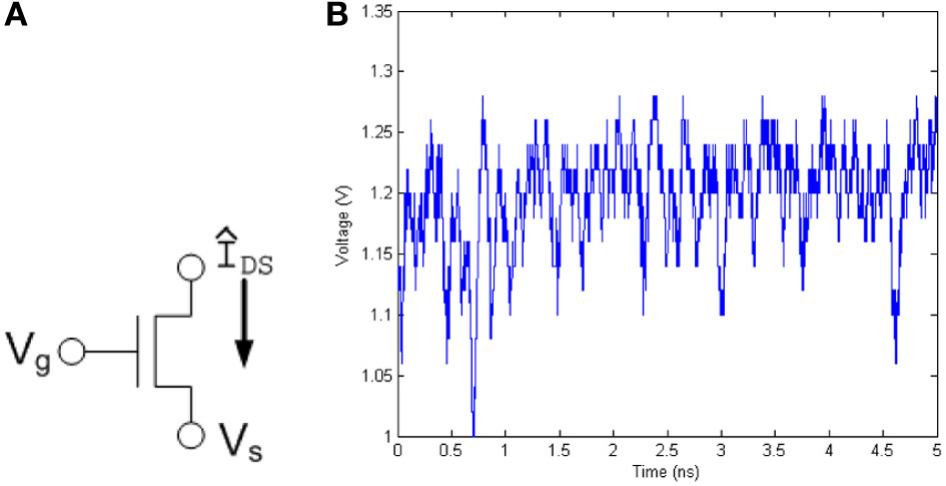
**(A)** The noise characteristics generated by a single transistor can be used as well-defined noise source. The current through and thus the voltage at the drain or source node of the transistor produces noise due to the thermal agitation of charge carriers. **(B)** Voltage measured from a transistor on 0.35 μm test chip.

The current through a transistor, and hence the voltage at the drain or source node of a transistor, shows the probabilistic thermal noise effect as shown in Figure 1, and can be used as the basic building block of any probabilistic function generator.

Thermal noise present in integrated circuits, also known as Johnson Noise, is generated by the natural thermal excitation of the electrons in a circuit. When modeled on a capacitive load, the root-mean-square voltage level of the thermal noise is given by $U_{T}=\sqrt{\frac{kT}{C}}$ where *k* is Boltzmann's constant, *T* is temperature, and *C* is the capacitance of the load. The likelihood of the voltage level of thermal noise is modeled as a Gaussian probability function and has an equivalent magnitude throughout its frequency spectrum and is thus known as white Gaussian noise (Kish, 2002).

**Figure 2:**
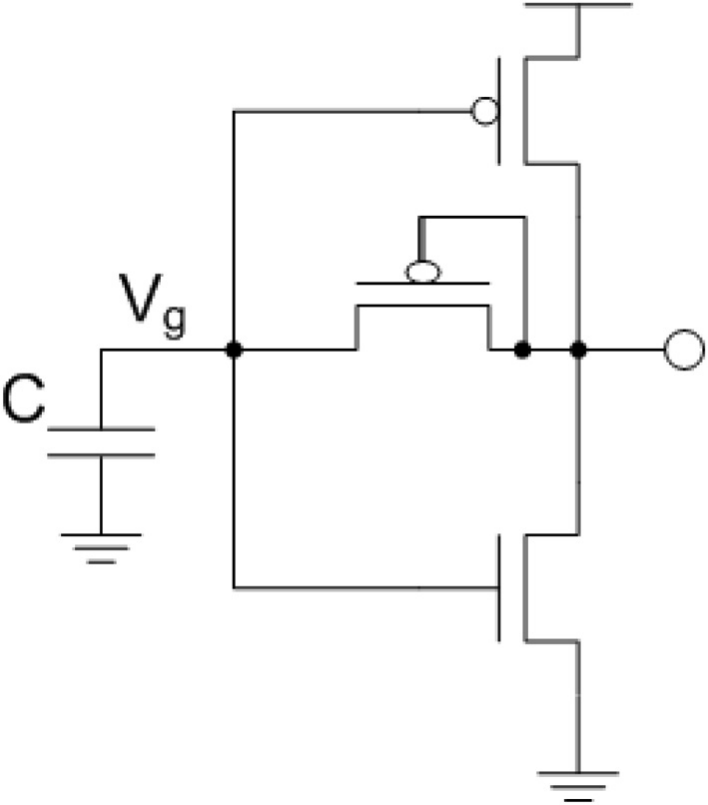
**Circuit used to take advantage of the well-defined thermal noise properties on a capacitor where the root-mean-square (RMS) voltage of the noise on a capacitor is $V_{n}=\sqrt{\frac{kT}{C}}$**. The thermal noise on this capacitor is used as the gate voltage for PMOS and NMOS transistors where a current with the same spectrum as the thermal noise is generated. This ultimately produces a voltage controlled current through the diode-connected transistor.

To take advantage of the well-defined properties of thermal noise trapped on a capacitor, the circuit in Figure 2A was developed. This circuit can be broken down into the circuit components seen in Figure 2B.

**Figure 3:**
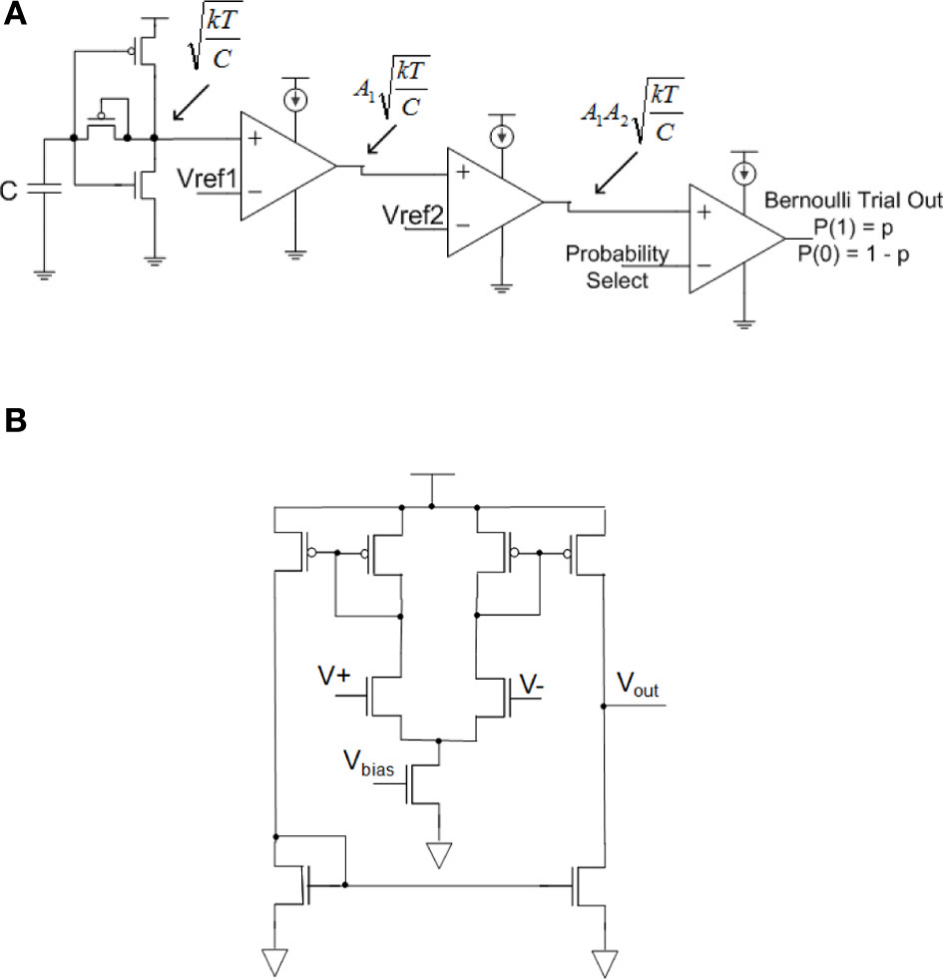
**Circuits for generating a Bernoulli random variable (1 with probability *p* and 0 with probability 1-*p*)**. **(A)** Dynamic Bernoulli Probability Circuit. Thermal noise is trapped on the capacitor, amplified twice through two operational transconductance amplifiers (OTA) with gain of *A*_*i*_ then put through a comparator with a probability select input. Note that these three OTA's are the same basic circuit programmed to different functionality. **(B)** Nine-transistor OTA used in **(A)** for amplifier and comparator circuits.

Thermal noise voltage on a 0.35 μm process, which is the process size used for testing in this paper, with capacitor values in the range of *C* = 500 fF has RMS noise level of roughly 100 μV. Even with a 100 mV supply, the thermal noise voltage that would cause a probabilistic digital bit flip is 1000σ down from supply, giving the probabilistic function designer limited options. Hence, in these experiments the thermal voltage signal was routed through two operational transconductance amplifiers (OTA) with gain *A*_*i*_. These circuits are shown in Figure 3.

**Figure 4:**
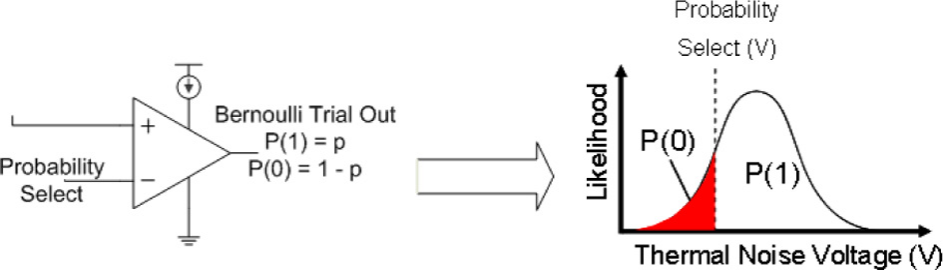
**The probability distribution function of thermal noise and the probability of generating a *P*(1) or *P*(0) in the Bernoulli variable generating circuit**. The probability of a 1 is the integral under the probability distribution function of the thermal noise from the comparator voltage to the supply voltage of the circuit, *V*_*dd*_.

By routing the amplified thermal noise signal through a comparator, and then comparing this signal to a user selectable voltage signal, a Bernoulli trial is created where the comparator outputs a digital “1” if the amplified thermal voltage signal is greater than the probability select and a “0” is output otherwise. A probability *p* can be set for the Bernoulli trial by setting the input voltage to the comparator such that it is less or more likely for a randomly varying thermal voltage to surpass this value, “Probability Select.” The integral from the input voltage to *V*_*dd*_ of the thermal noise function is the probability of a digital 1, and the probability of a digital 0 is the integral from ground to the input voltage. This concept is illustrated in Figure 4.

Note that the reconfigurable FPAA used to program this circuit has floating-gate controllable current biases such that they can be used to offset temperature effects.

**Figure 5:**
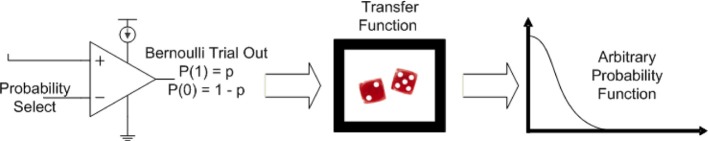
**A Bernoulli probability trial can be used to generate an arbitrary probability distribution with the correct transfer function**. This result will be taken advantage of to greatly increase the performance of dynamical systems in this paper.

A Bernoulli random variable is generated with a probability *p* dynamically selectable by the user using these techniques. A useful property of Bernoulli trials is that an arbitrary probability distribution can be created when used in large numbers (as the number of Bernoulli trials *N* → ∞) they can be used to create an arbitrary probability distribution. This phenomenon is illustrated in Figure 5.

An exponential random variable is generated from a Bernoulli variable in the following way. *X* is defined here as the number of Bernoulli trials needed to produce a success, and this variable *X* is exponentially distributed. For example, to require six coin flips to produce a heads is exponentially less likely than to require two flips to get a head, since this is nothing more than a standard geometric sequence. The shape of this exponential distribution is controlled by the probability *p* of the Bernoulli trials.

(1) $$Pr(X=k)={\left(1-p\right)}^{k-1}p$$

**Figure 6:**
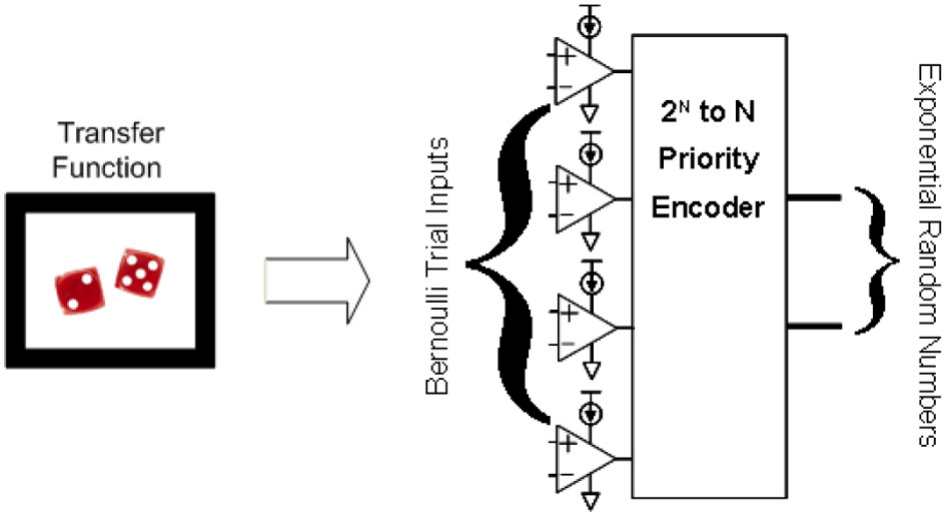
**Illustration of how to transform *N* Bernoulli trials into an exponential distribution**. Bernoulli trials put through a priority encoder as inputs results in an exponentially distributed probability function, where the shape of the exponential function can be tuned through the threshold inputs to the Bernoulli trials.

Figure 6 shows how these Bernoulli random variables are used to create an exponentially distributed number by being placed as inputs to a priority encoder. Recall that a priority encoder works by encoding the output to represent in binary which input was the first in priority order (from top to bottom for example) to be a 1. Also recall from Equation (1) that the number of Bernoulli trials needed to get a 1 is exponentially distributed. !The top Bernoulli trial in the figure is considered our first trial, the second from the top, our second trial etc. So the priority encoder in Figure 6 is encoding for us how many trials are needed to get a success (1), exactly our exponential distribution.

Using these methods, an exponentially distributed random number can be generated in two clock cycles, a vast improvement over software and other current methods, which will be explained in the next section.

### 2.2. Programming Bernoulli trials at telluride workshop

All of the above circuits from the previous section were conceived of, designed, built, measured, and compiled in about in the 2 weeks of the 2008 Telluride Neuromorphic workshop. This accomplishment is both a testament to the productivity that the Telluride Neuromorphic workshop allows as well as a testament to the quick prototyping capability of the FPAA.

The Telluride Neuromorphic workshop is unique in that some of the most brilliant minds in the country get together for an extended several week session dedicated to teaching, working with real hardware, and producing results such that students are completely immersed in a world class engineering environment, day and night, for 2–3 weeks.

The FPAA is analogous to the FPGA for logic circuits in that circuits can be conceived of, programmed onto the chip, and measured in the same day. The FPAA has a mature toolset where an analog designer can conceive of a circuit, such as during a Telluride lecture session on analog design, and can simply create a netlist. The FPAA tools automatically take this netlist and optimally compile it to the fabric using a bitstream to program the on-board floating gate devices to set switches allowing networks of active and passive devices, set current sources, bias currents, amplifier characteristics, and calibrate out device mismatch. A standard multi-meter is connected to the FPAA test board via built-for-test pinned out circuit leads. The multi-meter in this instance was connected back to the computer via GPIB that was producing the netlist to allow a full hardware in the loop programmable environment. Current toolsets are even more advanced allowing Simulink and other Simulink-like tools to build the circuit netlists.

### 2.3. Temperature invariant Bernoulli trials

The thermal noise circuits used to create Bernoulli trials shown in the previous section have the well known side effect that their accuracy is highly dependent on temperature. And although methods such as adjusting bias currents with temperature are available on the FPAA, we present a temperature invariant method here to address this potential variability in the Bernoulli trials presented previously. These chaos circuits were built as follow-up work to the Telluride workshop.

Chaos circuits were chosen to exemplify a more temperature invariant method. The model and explanation for the low-power chaos circuit used in this paper is first presented in Dudek and Juncu (2005).

A chaos circuit works by introducing a seed value to a non-linear “chaos map” circuit which is itself chaotic. The sample and hold circuit then captures a continuous voltage value for consumption by a stochastic algorithm. The chaos map from Dudek and Juncu (2005) was chosen because of its proven results, but also because it only requires nine transistors and is extremely energy efficient.

**Figure 7:**
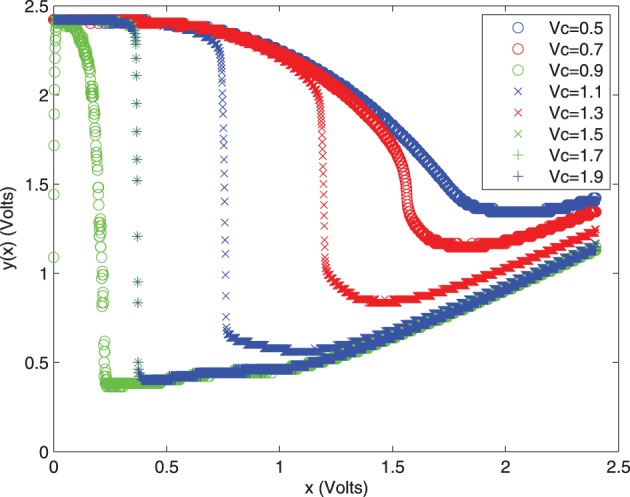
**Measured y(x) voltage vs. *V*_*c*_ for the chaos map circuit compiled on the FPAA**.

The resulting chaos map with a tunable control voltage to dictate the probability characteristics is shown in Figure 7.

While further reading may be needed to understand the chaos circuit map shown in Figure 7, this map is very close to the results expected as shown in the literature. The general idea is that a given output voltage will result in a random assignment to the chaos map, allowing us to generate random variables in a temperature invariant way. The idea is that this chaos map could be used in place of the thermal noise circuits should the designer be concerned about temperature.

These circuits all have something in common: they can be used to directly compute a stochastic function, cannot be compiled on a digital chip, and compute more efficiently than a digital system.

Next we show how the usefulness of these circuits in a dynamical system.

## 3. Gillespie's algorithm for stochastic computation

The previous findings are used to generate the results of a chemical and biological system using Gillespie's algorithm in this section. This section will also review the expense to calculate the trajectory of stochastic systems in software as a comparison. Gillespie's algorithm is a natively probabilistic algorithm that takes advantage of the naturally stochastic trajectories of molecular reactions (Gillespie, 1976), this algorithm is described below.

### 3.1. Gillespie's algorithm

1. Initialize. Set the initial number of each type of molecule in the system and time, *t* = 0.
2. For each reaction **i**, calculate the propensity function to obtain parameter value, *a*_*i*_.
3. For each **i**, generate a reaction time τ_*i*_ according to an exponential distribution with parameter *a*_*i*_.
4. Let μ be the reaction time whose time is least, τ_μ_.
5. Change the number of molecules to reflect execution of reaction μ. Set *t* = *t* + τ_μ_.
6. If initialized time or reaction constraints met, finished. If not, go to step 2.

We use complexity analysis, or big-Oh, analysis to analyze the algorithms here where *O*(*x*) gives an expression, *x*, that represents the worst case running time of the algorithm. Only algorithmic improvements in software have been made to computing Gillespie's algorithm, until this work, such as Gibson *et al*. who have improved the running time of the algorithm from *O*(*Er*) to *O*(*r + Elogr*) where *E* is the number of reaction events in the trajectory and *r* is the number of different reaction types (Gibson and Bruck, 1998). Several orders of magnitude improvement in energy efficiency and performance can be realized by computing the exponentially distributed random variable τ_*i*_ in hardware. Note that the big-Oh function does not change, just the way we implement this algorithm is much improved.

The generation of the exponentially distributed random number is the bottleneck of the algorithm, and the computational complexity of each step is calculated to show this. The metric used to judge computational cost is the number of clock cycles it takes to do a given calculation on a modern general purpose CPU as described in Patterson and Hennessy (2004).

A load instruction is used to initialize a variable in Step 1. With the best case with a multiple data fetch scheme such as in the cell processor, this requires a single computational step. The propensity function *a*_*i*_ in Step 2 is calculated by a floating point multiplication (FPMUL), which takes five computational steps in a modern processor per reaction (Gillespie, 1976; Patterson and Hennessy, 2004). All *r* reactions assuming δ FPMUL units are available takes $\frac{5r}{\delta }$ total computational steps. In Step 4, the minimum reaction time τ_μ_ takes *r* − 1 compare operations. Assuming δ ALU (compare) units are available, Step 4 takes $\frac{r-1}{\delta }$ computational steps. Step 5 involves *r* − 1 integer addition/subtraction operations taking again $\frac{r-1}{\delta }$ computational steps. Step 6 is an update in the program counter resulting in a single step. Step 3 is a key step where each τ_*i*_ according to an exponential distribution. Generating an exponentially distributed random number is complex and deserves a bit more treatment.

This is believed to be the first method to generate an exponentially distributed random number in hardware, and the random numbesr generated by other current methods is only *pseudo-random*. The Park–Miller algorithm on a modern advanced processor is the best known software method where a uniformly pseudo-random number is generated in 88 computational steps (Park and Miller, 1998; Patterson and Hennessy, 2004). The equation to transform a uniformly distributed random variable *U* to one with an exponential distribution *E* with parameter λ is shown in Equation (2).

(2) $$E=\frac{-\ln U}{\lambda }$$

The natural logarithm function, ln is extremely expensive, and even in the best case of computing this on a modern digital signal processor (DSP) takes 136 computational steps by itself according to Yang et al. (2002). Thus counting the FP multiply and the FP divide taking 5 steps and 32 steps, respectively (Patterson and Hennessy, 2004), it takes a total of 261 computational steps to generate a single exponentially distributed pseudo-random variable in software. Thus Step 3 alone takes 261*r* computational steps to generate τ_*i*_ for all *i* reactions. To review the number of computational steps for each part of the algorithm is shown below.

### 3.2. Computational steps in Gillespie's algorithm

**Table:** 

Algorithmic step	Computational steps
(1) Initialize.	1
(2) Multiply to find each a_*i*_.	$\frac{5r}{\delta }$
(3) Generate each τ_*i*_.	261r
(4) Find τ_μ_.	$\frac{r-1}{\delta }$
(5) Update.	$\frac{r-1}{\delta }$
(6) Go to step 2	1

Thus for a conservative value of δ = 2, generating each exponentially distributed τ_*i*_ in Step 3 takes approximately 98% of the computational steps for a single iteration of Gillespie's algorithm. Seen in this light, the problem of improving exponential random variable generation becomes quite an important one.

### 3.3. Expansion to any dynamical system

It has been shown in Gillespie (1976) and Kurtz (1972) that the trajectory of a chemical system consisting of *N* different reactant concentrations can be expressed as both a system of deterministic differential equations and a system of stochastic processes. For deeper understanding of Equations (3–5) that follows, the reader is encouraged to read these aforementioned references. This concept can be generalized to any dynamical system where the time evolution of a system of *N* state variables that has been described in a classical, state-based deterministic model as

$$\begin{array}{c} \frac{dx_{1}}{dt}=f_{1}(x_{1},x_{2},x_{3},...)\\ \frac{dx_{2}}{dt}=f_{2}(x_{1},x_{2},x_{3},...)\\ \ldots \end{array}$$

and in general as:

(3) $$\dot{X}=F(X)$$

This system can also be expressed as a stochastic, Markov process.

For the stochastic system, the probability that state variable *x*_μ_ is updated during the next time interval τ is assigned, *P*(τ, μ)*d*τ. More formally, this is the joint probability density function at time *t* expressing the probability that the next state update will occur in the differential time interval (*t* + τ, *t* + τ + *d*τ) and that this update will occur to state variable *x*_μ_ for μ = 1 … *N* and 0 ≤ τ < ∞.

Given probability *P*_0_(τ), the probability that no state-space update occurs in the interval (*t, t* + τ), and the probability, α_μ_ that an update to state *x*_μ_ will occur in the differential time interval (*t* + τ, *t* + τ + *d*τ) we have the general form for the joint probability function:

(4) $$P(\tau ,\mu )d\tau =P_{0}(\tau )\cdot \alpha_{\mu }d\tau$$

Note that α_μ_ is based on the state of the system **X**. Also note that determining α_μ_ is the critical factor in determining the Markov process representation and no general method for this is given here. In the chemical system example, α_μ_ is the probability that reaction *R*_μ_ is going to occur in the differential time interval and is a function of the number of each type of molecule currently in the system. The probability that more than one state update will occur during the differential time interval is shown to be small and thus ignored (Kurtz, 1972; Gillespie, 1976). Finally some function *g*_μ_ must be given describing the update to state variable *x*_μ_ once an update occurs. We then have the stochastic, Markov process defined for the system:

(5) Pr[X(t+τ+dτ)=G(X(t))∣X(t)]=P(τ,μ)

Note that this formulation does not make the assumption that infinitesimal *d*τ need be approximated by a finite time step Δτ, which is a source of error in many Monte Carlo formulations.

To solve this system using computational methods, random numbers are generated according to the probability distributions described by Equation (5). No matter what dynamical system is involved, exponentially distributed random numbers will always be needed. To calculate *P*_0_(τ) from Equation (4), we break the interval (*t, t* + τ) into *K* subintervals of equal length ϵ = τ/*K*, and calculate the probability that no state update occurs in the first ϵ subinterval (*t, t* + ϵ) which is:

(6) $$\prod_{i\;=\;1}^{N}\left[1-\alpha_{i}\epsilon +o(\epsilon )\right]=1-\sum_{i\;=\;1}^{N}\alpha_{i}\epsilon +o(\epsilon )$$

This probability is equal for every subinterval (*t, t* + 2ϵ), (*t, t* + 3ϵ), and so on. Thus the probability *P*_0_(τ) for all *K* subintervals is:

(7) $$P_{0}(\tau )=\underset{K\to \infty }{\lim}{\left[1-\sum_{i\;=\;1}^{N}\alpha_{i}\epsilon +o(\epsilon )\right]}^{K}$$

(8) $$\;=\underset{K\to \infty }{\lim}{\left[1-\sum_{i\;=\;1}^{N}\alpha_{i}\tau /K+o(\tau /K)\right]}^{K}$$

Where *o*(ϵ) is the probability that more than one event occurs in the time interval ϵ. Following the analysis in Gillespie (1976), we assume as our incremental time interval goes to zero our function *o*(ϵ) → 0 as well. With *o*(τ/*K*) → 0 we are left with the probabilistic, exponential function in Equation (9).

(9) $$P_{0}(\tau )=exp\left[-\sum_{i\;=\;1}^{N}\alpha_{i}\tau \right]$$

Thus we prove how this work can be extended to any dynamical system.

## 4. Reconfigurable analog hardware for stochastic computation

**Figure 8:**
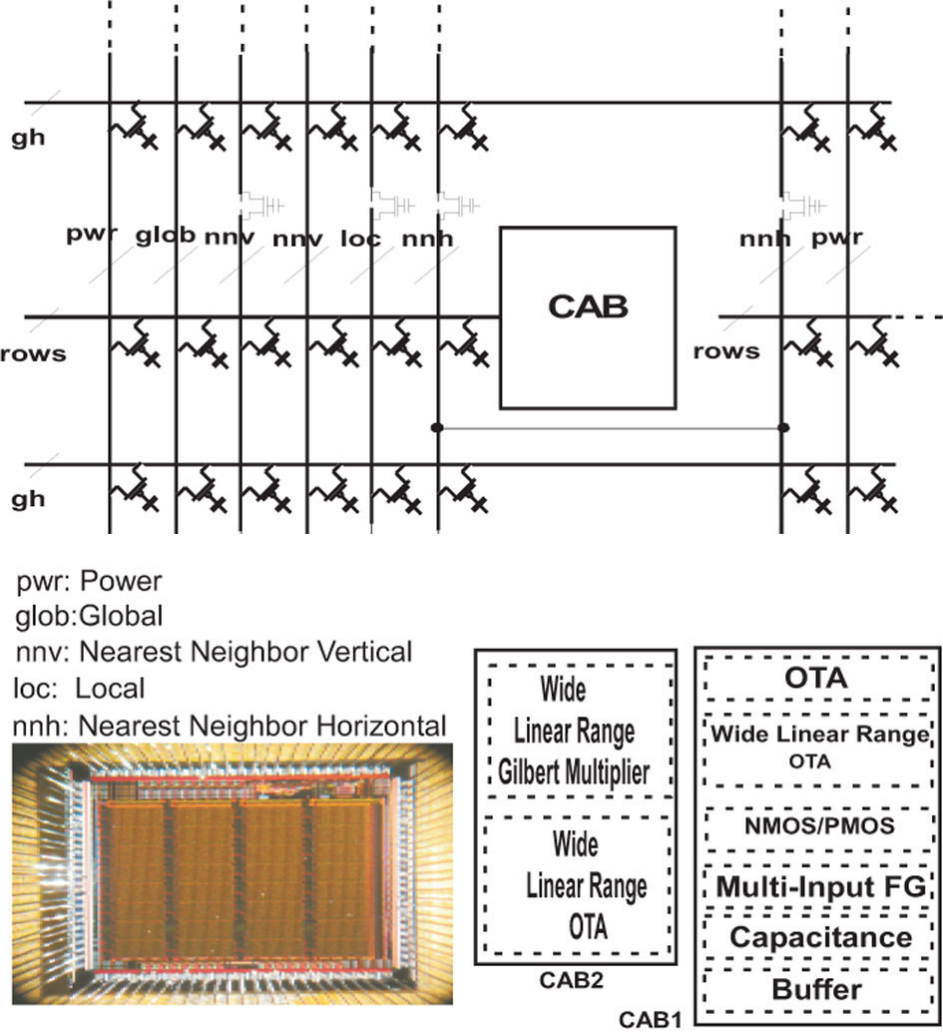
**Micrograph of the Reconfigurable Analog Signal Processor (RASP), also referred to the Field Programmable Analog Array (FPAA)**. The circuits were compiled onto this device. Computational Analog Blocks (CAB) populate the chip where both the computational elements and the routing is configured via floating gates. Buffers, capacitors, transmission gates, NMOS, PMOS, floating gates, current biases, adaptive amplifiers, and analog multiply arrays are all available and fully programmable on the chip. Development is shared by many in the CADSP group at Georgia Tech.

These complex probability functions are generated on a reconfigurable platform, reviewed in this section. More specifically, a dynamic *Bernoulli Trial* generator is illustrated on chip. This novel method involves using the reconfigurable analog signal processor (RASP) chip that was recently introduced (Basu et al., 2008). This device allows one to go from concept to full functionality for analog circuits in a matter of weeks or even days instead of months or years for large-scale, integrated circuits. The design presented went from concept to measured hardware in a matter of 2 weeks. The other useful feature is that many FPGA type architectures allow a designer to build a subset of the possible circuits available with the RASP. Circuits such probabilistic function generators could not be produced on strictly digital reconfigurable architectures although digital designs can be built on the RASP. The RASP chip is shown in Figure 8.

### 4.1. Stochastic circuit architecture

**Figure 9:**
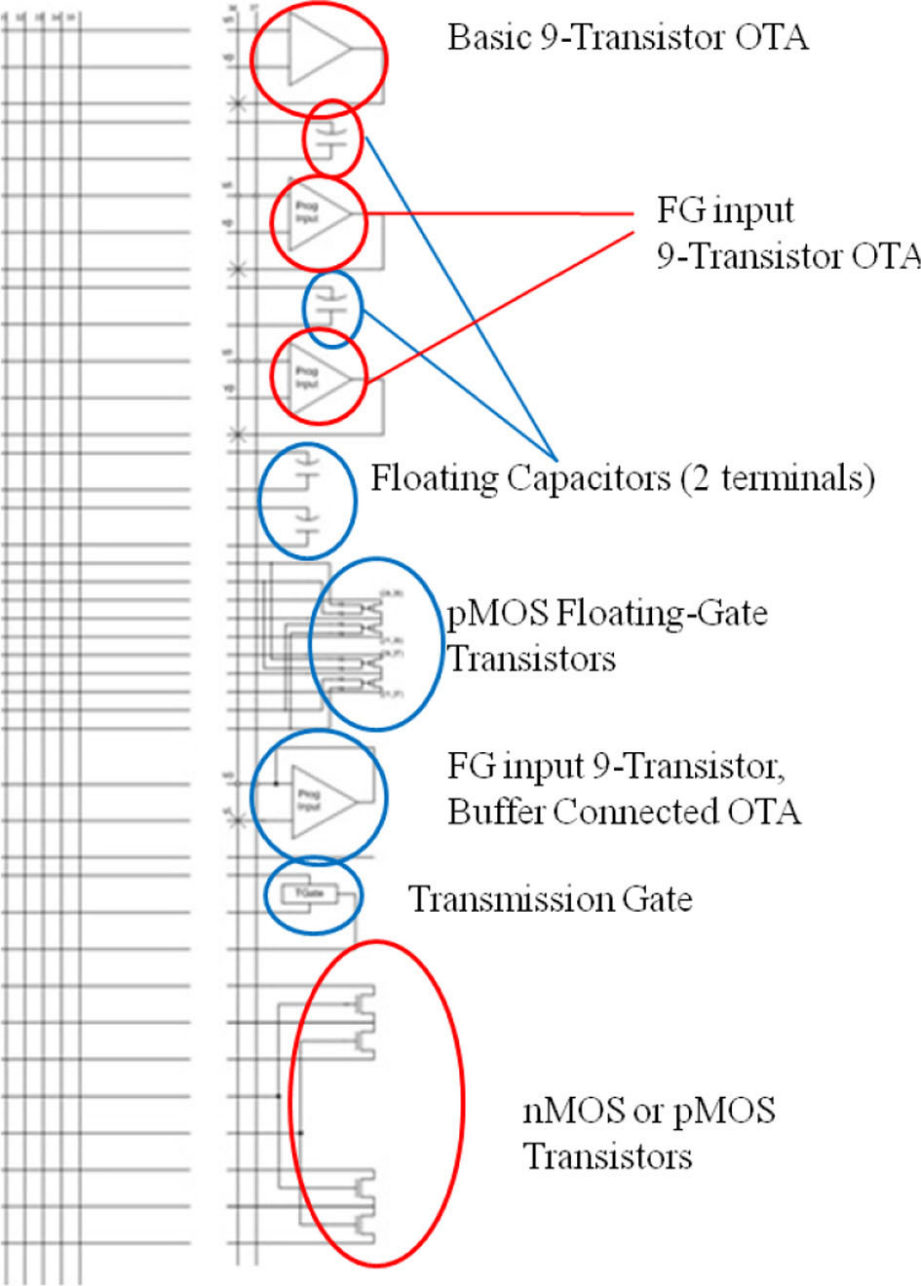
**The typical routing structure and available analog computational elements in one of the 32 computational analog blocks (CABs) present on the RASP chip**. The elements used in the present design are highlighted in red. Alternating CABs have NMOS transistors and PMOS as the bottom CAB element, and although only three of the MOSFETs are used in our circuit from Figure 3, instead of the four that are circled, this is meant to show several different combinations of these four transistors can be used to make the three transistor circuit. The top triangle elements are an OTAs showing the two inputs and output being routed back into the fabric which is a mesh of lines with switches able to connect any two lines that cross in the mesh. The element below the OTAs is a capacitor and the bottom elements circled are NMOS and PMOS available to be routed.

The macroarchitecture and details of the algorithmic implementation via Bernoulli trials and how this is built on the RASP chip is explored here. The RASP has 32 reconfigurable, computational analog blocks (CABs). The elements of a CAB and the elements that are used in this design are shown in Figure 9.

An array of up to 32 Bernoulli trials can be calculated simultaneously on a single RASP device. New versions of the RASP have been fabricated in 350, 130, and 45 nm; an entire family of RASP 2.9 chip variants exist for different applications spaces, which allow as much as 10X this number of Bernoulli trials, and this scales with Moore's law. The RASP chipset and accompanying tools also have the ability to be linked together easily for a multi-core RASP chipset should more Bernoulli generators be needed. The RASP chipset is useful for a proof of concept here. Since each Bernoulli generator only takes 30 transistors, many thousands of these circuits could be built in custom hardware if needed.

## 5. Chip measurements and experimental results

To gather data, the Probability Select line described in Figure 3A and the output producing the random numbers were routed to the primary inputs/outputs of the chip. A 40-channel, 14-bit digital-to-analog-converter (DAC) chip was interfaced with the RASP chip on a printed circuit board, which we used as our testing apparatus, so that any arbitrary voltage could be input to the Probability Select line. This chip is 40 channel in the sense that there are 40 independent outputs of the DAC. The outputs of the RASP chip were connected to oscilloscope probes so that the noise spectrum and random numbers could be captured.

**Figure 10:**
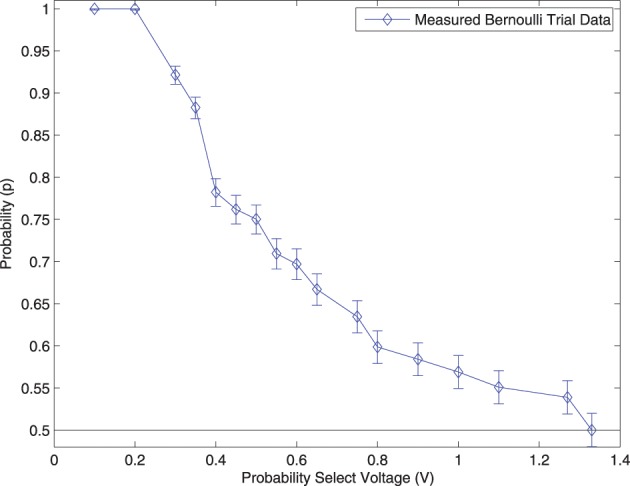
**Measurements from the output of the Dynamic Bernoulli Probability Circuit shown in Figure 3 were taken**. The “Probability Select” voltage was adjusted from 0.1 volts up to 1.4 volts and the resulting probability of a digital “1” being produced was recorded with a 95% confidence interval. Measurements were only taken down to *p* = 0.5 since a Bernoulli trial is symmetric about this value.

The distribution of the Bernoulli trial circuits were measured in the following way: 2500 random numbers were captured at each Probability Select voltage. The number of successes was divided by the total number of samples captured at each voltage to calculate the probability of a Bernoulli success (probability of randomly generating a 1). The results are shown in Figure 10.

**Figure 11:**
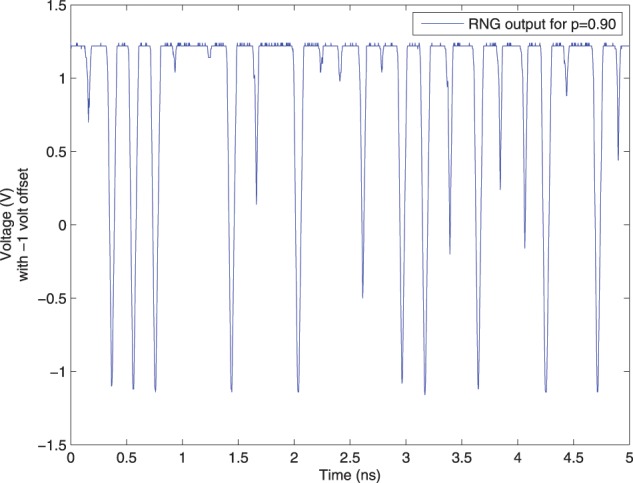
**Voltage output from Dynamic Bernoulli Probability Circuit also called a random number generator (RNG) circuit as labeled in the graph, corresponding to the output out of the third (final) OTA circuit from Figure 3**. A 1 volt offset was arbitrarily chosen for the comparator, but other voltage offset values than this were anecdotally observed to have undesired noise effects resulting in spurious switching at the output of the comparator. The noise amplifiers, and thus comparators, were observed to switch at approximately 208 ps as a maximum rate, thus independent Bernoulli variables could be produced at most at this time period in this particular device. A digital “1” is produced with probability *p* = 0.90 and “0” with 1 − *p* = 0.10. When the voltage is above the threshold $V_{out}>\frac{V_{dd}-V_{ss}}{2}$ it is considered a digital “1” and otherwise a “0,” where the threshold happens to be 0 volts in this case. The samples in this circuit change much faster than can be consumed, and thus random samples are taken from the output of this circuit at a slower rate than the rate at which it changes state, but preserving randomness.

An example of a voltage signal from the Dynamic Bernoulli Probability Circuit producing a digital “1” with probability *p* = 0.90 is shown in Figure 11. The voltage signal was recorded via on-chip measurement circuits and transmitted to a PC through a USB connection to the chipset.

**Figure 12:**
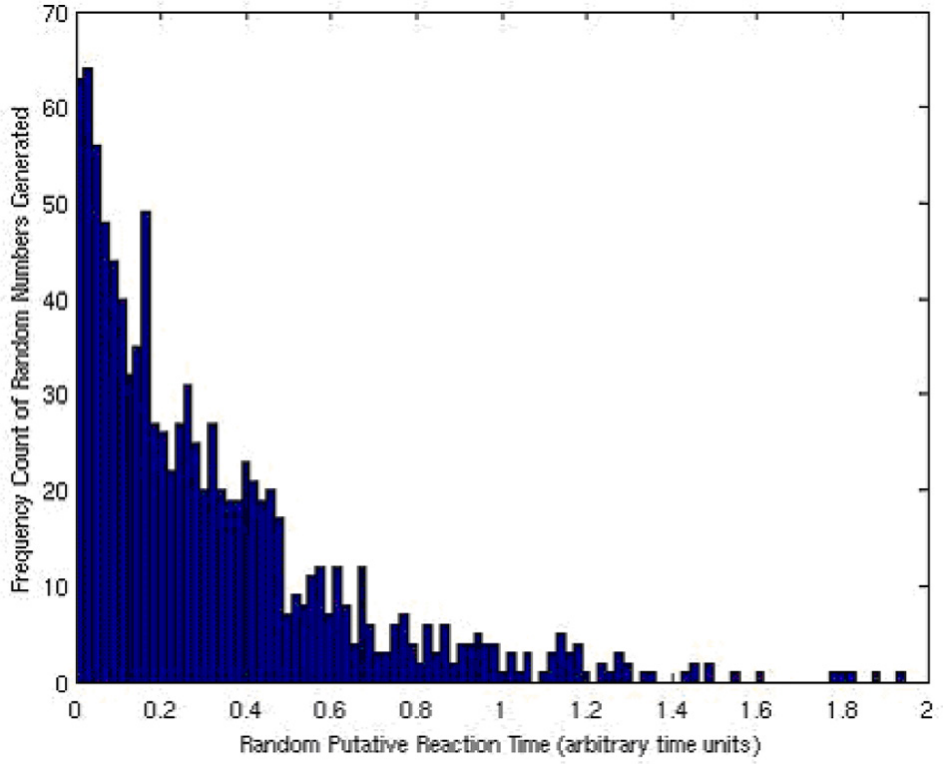
**The histogram (and thus a scaled probability distribution function) of the exponentially distributed Gillespie reaction times, τ_*i*_'s, generated**.

The array of Bernoulli trials was encoded and the exponential distribution of reaction times, τ_*i*_, was generated. The resulting distribution is what one would expect and matches a true, exponential distribution as shown in Figure 12.

### 5.1. Validation of randomness

A *probabilistic* output and a *random* output are differing concepts, and the ability to control this difference is the strength of the proposed circuits. They are linked together and defined through Shannon's entropy (Shannon, 1949). Formally, entropy and thus the randomness of a function are defined by *H* in Equations (10, 11).

Let

(10) $$H_{n}=-\frac{1}{n}\sum_{i,j,...,s}p(i,j,...,s)\log_{2}p(i,j,...,s)$$

Then entropy is

(11) $$H=\underset{n\to \infty }{\lim}H_{n}$$

where *p*(*i, j, …, s*) is the probability of the sequence of symbols *i, j, …, s*, and the sum is over all sequences of *n* symbols.

By the same definition, a function exhibits the *most randomness* if *H* is maximized, which occurs when all output sequences are equally likely, or equivalently, if all possible outputs have an equal probability of occurring (Shannon, 1949). From this work, a function is defined as *random* if all outputs have a uniform probability of occurring. Conversely, we define a function as *probabilistic* if the function has an entropy 0 < *H* < log_2_ *n*.

There exist statistical measures of randomness, developed by the National Institute of Standards and Technology (NIST), in the form of a suite consisting of 16 independent tests to measure *how random* a sequence of numbers truly is Random Number Generation and Testing (2014). However, these tests only measure the performance of *random* functions and not *probabilistic* ones such as the circuits presented in this work, although *random* number generation (when *p* = 0.5) is a subset function of these circuits.

Each of the tests are measured on a scale from 0 to 1, where a “passing” mark is considered > 0.93 and higher marks indicate a higher quality sequence. For a random output *p* = 0.5 these circuits with thermal noise as the source of randomness passed all but the “Overlapping Template Matching Test” and the “Lempel-Ziv Complexity Test” and even these two tests received high marks > 0.80. They also perform consistently better than the software generated, Park–Miller *psuedo-random* numbers used by most algorithms, which failed half the tests in the suite with some failing badly <0.10 (Chakrapani et al., 2006).

## 6. Conclusions and future directions

It was shown in section 3 that to generate an exponentially distributed random variable in software takes a minimum of 261 computational steps with the Park Miller algorithm. And with hardware random number generators shown in previous microprocessor works, only uniformly random numbers were available. Bernoulli trials are generated here in hardware with a single computational step, and an exponentially distributed random number is generated with two computational steps.

Because of the high gain of our amplifiers, the thermal noise distribution used to generate probabilistic distributions with our hardware is extremely sensitive to perturbations such as ambient electrostatic interactions, device variations, and changes in ambient temperature. Environment invariant chaos circuis were compiled and measured to mitigate these concerns. Because the Bernoulli trial circuits presented here can be controlled via a programmable input signal, software calibration can be done to mitigate these concerns as well.

An estimated performance increase of approximately 130X is realized based on measured results to generate exponentially distributed random numbers. With the assumption used in section 3 that generating exponential random variables takes up 98% of the computation time of a single iteration through Gillespie's algorithm, our system could potentially speed up the calculation of the trajectory of this algorithm by approximately 127X.

Further is was shown that these performance increases via hardware generated probabilistic distributions can be applied to *any dynamical system* and possibly have a much wider impact than the field of biological computations.

Such a method to increase computational efficiency by two orders of magnitude is believed to be widely useful in calculating biological, statistical, or quantum mechanical systems. The search for meta-materials new medicines, or any other host of applications could benefit. Future directions for this specific work include attempting to hardware accelerate quantum algorithms and venturing into the world of software defined analog radios.

### Conflict of interest statement

The authors declare that the research was conducted in the absence of any commercial or financial relationships that could be construed as a potential conflict of interest.
